# Combined Use of Non-Invasive and Micro-Invasive Analytical Investigations to Understand the State of Conservation and the Causes of Degradation of *I Tesori del Mare* (1901) by Plinio Nomellini

**DOI:** 10.3390/mps5030052

**Published:** 2022-06-18

**Authors:** Andrea Macchia, Chiara Biribicchi, Laura Rivaroli, Hélène Aureli, Eleonora Cerafogli, Irene Angela Colasanti, Paola Carnazza, Giuseppe Demasi, Mauro Francesco La Russa

**Affiliations:** 1 Department of Biology, Ecology and Earth Sciences (DiBEST), University of Calabria, Via Pietro Bucci, 87036 Arcavacata, Italy; andrea.macchia@uniroma1.it (A.M.); mlarussa@unical.it (M.F.L.R.); 2 YOCOCU (Youth in Conservation of Cultural Heritage), Via T. Tasso 108, 00185 Rome, Italy; heleneaureli94@gmail.com (H.A.); cerafogli.1559339@studenti.uniroma1.it (E.C.); colasanti.1748988@studenti.uniroma1.it (I.A.C.); 3 Department of Earth Sciences, University of Rome La Sapienza, P.le Aldo Moro 5, 00185 Rome, Italy; 4 Department of Architecture, University of Bologna, Viale del Risorgimento 2, 40126 Bologna, Italy; laura.rivaroli2@unibo.it; 5 Galleria Nazionale d’Arte Moderna e Contemporanea, Viale Delle Belle Arti 131, 00197 Rome, Italy; paola.carnazza@beniculturali.it; 6 Independent Researcher, Via Arci 9, 02032 Fara In Sabina, Italy; giuseppedemasi11@yahoo.it

**Keywords:** FT-IR ATR, Raman, SEM/EDS, multispectral imaging, zinc white, zinc stearate, metal soaps, acrylic resin, oil painting, cultural heritage

## Abstract

In this study, the investigation of the oil painting on canvas *I Tesori del Mare* made by Plinio Nomellini in 1901 is presented. The aim of the research was threefold: the examination of the state of conservation in view of the restoration treatment, together with the identification of the causes of degradation and the study of the artistic technique. During the years, the artwork underwent several cleaning and fixing interventions, resulting in a patchy appearance of the surface. Nevertheless, the presence of consistent liftings persists, while the protective coating shows uneven chromatic alteration, both requiring further analysis. Multispectral imaging allowed for better visualization of the figuration’s structure and the restored areas. The combined use of Raman spectroscopy, Fourier Transform Infrared spectroscopy in the Attenuated Total Reflection mode (FT-IR ATR), and Scanning Electron Microscopy coupled with an Energy Dispersive Spectroscopy (SEM/EDS) enabled better understanding of the stratigraphy through the identification of some pigments, the binder, and the aged varnish layer on the top. SEM/EDS highlighted the presence of zinc in both the ground layer and the paint layers. Furthermore, FT-IR ATR spectroscopy showed peaks related to metal soaps such as zinc stearate, which are known to cause severe delamination of the paint layers, explaining the recurring lifting issues. Eventually, the varnish layer was found to be acrylic resin, presumably mixed with varnishes applied in past restoration treatments.

## 1. Introduction

**Figure 1:**
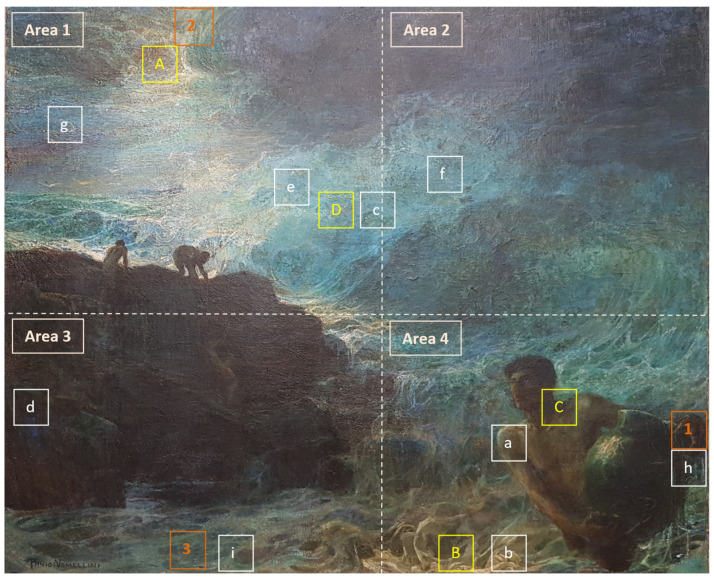
Plinio Nomellini, *I Tesori del Mare* (1901), 127 × 108 cm, Galleria Nazionale d’Arte Moderna e Contemporanea, Rome (IT). Image before the intervention with the indication of the investigated areas: sampling areas (1, 2, 3); acquisitions with DinoLite optical microscope (a–i); areas analyzed using Raman spectrometer (A–D); areas examined using Multispectral Imaging (Area 1–Area 4).

Plinio Nomellini was one of the greatest representatives of the Divisionist group. Starting from Macchiaioli’s realistic style and following Giovanni Fattori’s example, Nomellini’s language changed greatly when he moved to Genoa in 1890 [1,2]. During these years, Nomellini was influenced by Pellizza da Volpedo, engaging in social art halfway between the naturalist and divisionist styles (see *I mattonai*, 1889) [3]. Inspired by Camille Pissarro, he experienced a transition from Macchiaioli’s style to impressionist forms of expression, until flowing into an instinctive pointillism, made of points and small lines (see *Il Golfo di Genova*, 1891; *Il naufrago*, 1893; *La diana al lavoro*, 1893) [2,3,4]. Influenced by the atmosphere and the light effects of Genoa’s sea, Nomellini abandoned the realism and dark palette that characterized the early period, embracing clear luminosity and more idyllic atmospheres [1]. The restlessness of the early 20th century in Europe influenced Nomellini’s production in 1900–1907, showing mythical, philosophical, and literary Symbolism (see *Ditirambo*, 1905) [4]. The artist adopted a new material and decorative pointillism with iridescent light effects coming from the sea and sky in his paintings. The oil painting *I Tesori del mare* (1901) shows the typical features of the paintings made by the artist between 1900 and 1907, namely, the symbolistic dimension of the iconography and the peculiar light effects given by the sea waves. Even though a thorough investigation of Nomellini’s artistic technique has never been carried out, analyses performed on other works by the artist showed changes in the palette choice over the years [5,6,7]. In *Piazza Caricamento a Genova* (1891), the colors still seem to belong to a 19th century palette. Newly manufactured pigments are missing. A clear change in the selection of colors probably occurred in the following years, as can be seen from the analysis carried out on *Sole sulla brina* (1905–1910) and *L’imbarco dei Mille a Quarto* (1911) [6]. Here, new pigments were detected, such as cobalt violet, cadmium red, chromium oxide green, and cerulean blue, which were introduced in the market respectively in 1859, 1907, approximately 1859, and 1860 [6,7,8]. In this framework, the multi-analytical investigation performed on the oil painting on canvas *I Tesori del Mare* (1901) aimed at both defining the state of conservation of the artwork and understanding the causes of deterioration through the analysis of the stratigraphy (Figure 1). During the 20th century, the painting underwent several restoration treatments, involving cleaning interventions and the application of new protective coatings in 1917 and 1950, fixing of the paint layer in 1950 and 1971, and re-lining in 1971, as attested by the documentation at Galleria Nazionale d’Arte Moderna e Contemporanea in Rome (Italy). At the time of the analysis, the artwork showed aged layers of a coating material, incoherent sediments of dust, and lifting of the paint layer, mostly evident in the central area. Therefore, the implemented multi-analytical approach allowed the acquiring of significant elements to identify the materials and the structure of the stratigraphy, also providing substantial information to define the need for cleaning treatment of the artwork [9,10]. Multispectral imaging, Raman spectroscopy, and DinoLite acquisitions were carried out in the Restoration Laboratory of the Galleria Nazionale d’Arte Moderna e Contemporanea in Rome, enabling the selection of the more significative sampling areas to perform micro-invasive analyses, and providing relevant data regarding the varnish layer and the artistic technique (Figure 1). However, given the aims of the research, non-invasive analyses alone could not provide sufficiently meaningful data [11]. Therefore, micro-invasive analyses, i.e., Scanning Electron Microscopy coupled with an Energy Dispersive microanalytical System (SEM/EDS) and Fourier Transform Infrared Spectroscopy (FT-IR) in Attenuated Total Reflectance (ATR) mode, were carried out at the YOCOCU APS laboratory in Rome (Figure 1).

## 2. Materials and Methods

Multispectral imaging was performed using infrared (IR), ultraviolet (UV), and visible (VIS) light sources. UV fluorescence enabled the acquisition of relevant information about the surface of the artwork, such as the eventual presence of the varnish, previous retouches, or non-original layers. Near- and Mid-Infrared (NIR-MIR) spectrum bands were used to examine the underneath layers of the pictorial film and to verify the presence of restored areas through the analysis of IR-active materials. The analysis was carried out using Madatec multispectral system, which consists of a full-spectrum Samsung NX500 Digital Camera (28.2 MP BSI CMOS) and Madatec spotlights with 365 nm (UV) wavelength. Images of the induced fluorescence were taken using the Yellow 495 52 mm F-PRO MRC 022 filter to reduce the blue component of the UV spotlight, thus better highlighting possible fluorescence effects [9,10,12]. An IR filter at 750 nm was used to acquire IR images. Portable digital microscope DinoLite AM411-FVW enabled further examination of the surface morphology at different magnifications—from 40× to 220×—and illumination modes—visible light (VIS), raking light (VIS-RAD), and ultraviolet light (UV). Raman spectroscopy was used to gain information about the molecular structure of the materials constituting the artwork, through the investigation of the spectral region of intra- and intermolecular vibrations. Raman spectra were collected using Oceanhood’s portable 785 nm Raman Spectrometer RK785-I instrument, using a 785 nm laser with power ranging from 0–150 mW coupled with a portable DinoLite digital microscope for beam spot analysis (500 μm spot). The typical resolution of the spectrometer is 4 cm^−1^.

Micro-samples were taken from the painting, selecting significant while peripheral regions to preserve the integrity of the main areas (Figure 1). Three samples, including the entire stratigraphy or part of it, were collected to analyze the backside of the canvas, the ground layer, and the paint layers. Samples 1 and 3 include the entire stratigraphy from the original canvas to the varnish layer, which unevenly covers the paint layer, keeping it partially exposed. Sample 2 consists of all the layers from the original canvas to the paint layer. It was taken from the edge of the painting, allowing the direct analysis of the paint, where no overlapping layers can be detected. Samples were examined through digital microscopy and then with SEM/EDS to characterize the stratigraphy and the composition of the various layers at the elemental level. A Tescam microscope with Oxford EDS was used. The analysis allowed the examination of samples without pre-treatment, due to its capability of working even in non-high vacuum or in high vacuum conditions with low beam intensity. For the investigation of *I Tesori del mare*, we selected the low vacuum mode (15 Pa) with a 30 kV voltage of electron acceleration without pre-treating the sample and using the detector for backscattered electrons. FT-IR ATR spectroscopy was performed to gain information about the varnish, the paint layers, the ground layers, and the canvas. Spectra were collected on samples without pre-treatment by directly placing them on the diamond of the ATR due to the samples’ dimensions and to avoid preparation steps. FT-IR spectra were acquired using the Nicolet Summit FT-IR spectrometer and the Everest™ Diamond ATR accessory with a resolution of 8 cm^−1^, and 32 scans were performed on each sample. Then, collected spectra were evaluated using the instrument library, scientific literature, and the database edited by Vahur et al. [13,14].

## 3. Results

### 3.1. Multispectral Imaging

**Figure 2:**
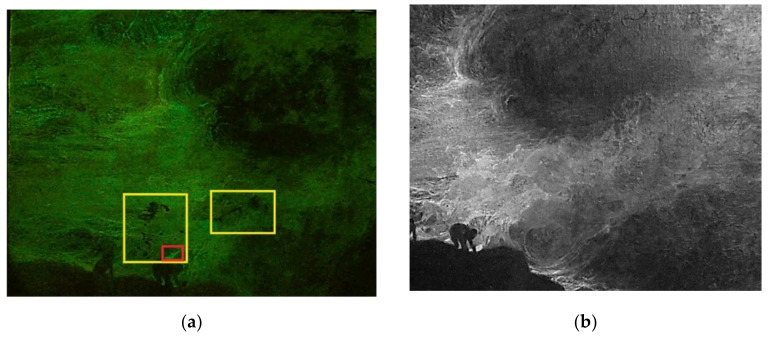

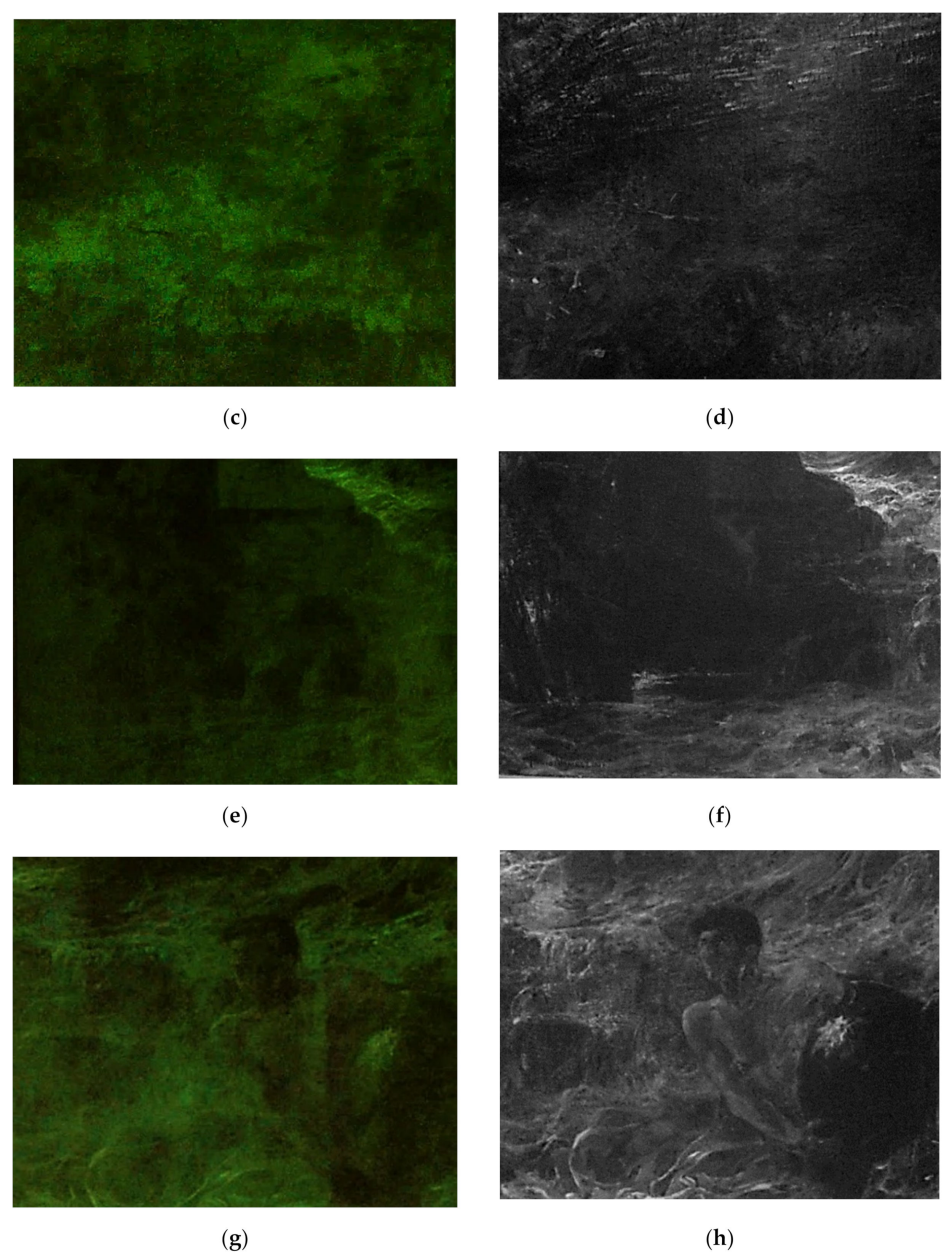
Multispectral imaging: UV light, using the Yellow 495 52 mm F-PRO MRC 022 filter (examples of the restored areas are highlighted in yellow, color gaps in red); IR images using the IR filter at 750 nm. Area n° 1: UV (**a**) and IR (**b**). Area n° 2: UV (**c**) and IR (**d**). Area n° 3: UV (**e**) and IR (**f**). Area n° 4: UV (**g**) and IR (**h**).

Due to the dimension of the artwork, the surface was ideally divided into four squares—i.e., areas n° 1, 2, 3, and 4—for careful examination through multispectral analysis (Figure 1). The acquired images show different fluorescence emission, varying from low to high fluorescence. The information collected through multispectral imaging allows the supposition that the highly UV-fluorescence-responsiveness should be ascribed to the presence of coating and fixing materials. Indeed, these areas were fixed, cleaned, and varnished during past restoration treatments. In addition, infrared images (IR) allowed for clearer visualization of the figure’s shapes, highlighting the differences among the different areas under IR radiation (Figure 2b,d,f,h).

Area n° 1 corresponds to the top-left quarter of the painting, including part of the cloudy sky and two figures climbing on the cliffs. The sky shows inhomogeneous fluorescence emission induced by UV radiation, proving the presence of an unevenly spread varnish layer (Figure 2a). In addition, UV radiation highlights the presence of dark spots differing from the surrounding area, which are not related to the figuration (Figure 2a). Due to their appearance, dark spots can be ascribed to past restoration treatments with a certain degree of confidence. Eventually, lighter fluorescence was detected in different areas, such as the one above the figure on the right side, where the ground layer is exposed due to a color gap (Figure 2a).

Area n° 2 corresponds to the top-right quarter of the artwork, which includes the cloudy sky. The analysis in visible light highlighted the presence of lighter colors presumably corresponding to past restoration treatments performed on detached or missing paint layers. Once again in this area, the fluorescence induced by UV radiation appears to be inhomogeneous, while IR radiation highlights the presence of small white spots above the rocks corresponding to color gaps, and larger light-grey areas corresponding to chromatic reintegration (Figure 2c,d).

Area n° 3 includes part of the turbulent sea and the bottom area of the cliffs on which the waves lush out. Multispectral imaging in visible light unearthed the presence of the author’s black signature in block letters on the left-bottom area (Figure 1). This shows the same fluorescence detected in other areas of the painting, underlining the application of an unevenly spread varnish (Figure 2e). Once again, the IR image made it possible to precisely recognize both the underlying structure of the figuration and the white spots which appear to be the result of past restoration treatments (Figure 2f). Specifically, the two figures climbing the cliffs are well delineated by IR radiation, unearthing details that would not have been noticeable otherwise.

Area n° 4 depicts the core of the painting, namely, a figure holding a heavy vessel which is partially immersed in the sea. Observations in visible light showed widespread cracking and small lifting of the paint layer in the upper area. A small black crack can be seen at the bottom of the vase, while a white reintegration is visible in the lower corner of the painting. UV light induces intense and bright fluorescence, once again highlighting uneven distribution of the varnish over the entire area (Figure 2g).

Once again, the infrared image allowed clearer definition of the figures’ shapes, also outlining the presence of liftings of the paint layer and small color gaps which are spread over the entire area (Figure 2h).

**Figure 3:**
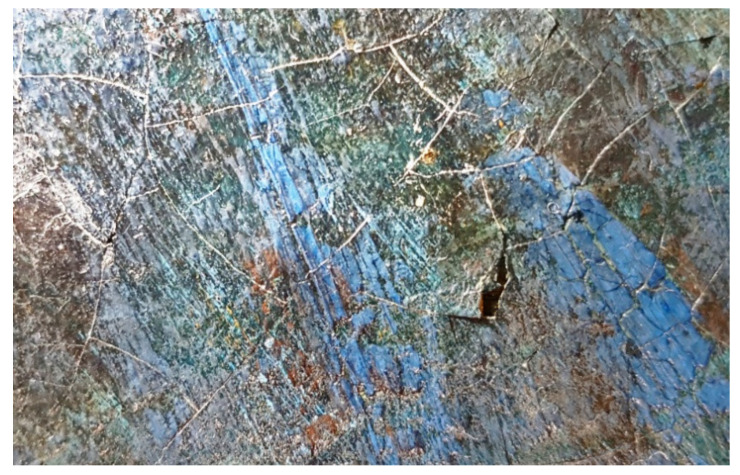
Detail of a lifted and cracked area in VIS-RAD light.

Eventually, images acquired in VIS raking light further brought out the presence of liftings, cracking, and gaps that are spread over the entire surface, highlighting the inner brittleness of the painting (Figure 3).

### 3.2. DinoLite Portable Digital Microscope

The Dino-lite portable digital microscope—in VIS and UV light—was used to acquire images at a high level of detail, thus obtaining further information on the nature and the morphology of both the varnish and the paint layers and better examination of the lifted areas (Figure 41a,1b). The investigation allowed more detailed understanding of the texture and the condition of both the original layers and the restored areas, also guiding the selection of the most representative sampling areas.

**Figure 5:**
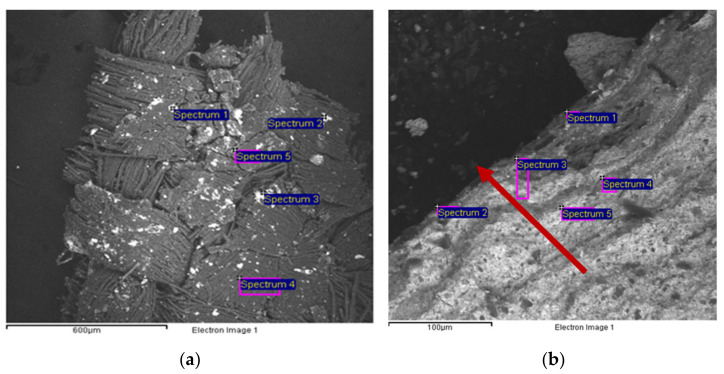
SEM images showing sample 1, consisting of the whole stratigraphy from the original canvas to the varnish layer. Image of the canvas (backside) (**a**) with the indication of the analyzed areas corresponding to the results in Table 1. Image of the layers from the preparation layer (arrowhead) to the paint layer on the top (starting point of the arrow) (**b**) with the indication of the analyzed areas corresponding to the results in Table 2.

At a microscopic level, the fluorescence emitted by the top-layer is more noticeable. Indeed, the paint layer seems to be heterogeneously covered by a resinous varnish which shows yellow-green fluorescence when irradiated by UV light (Figure 42a–5b).

**Figure 6:**
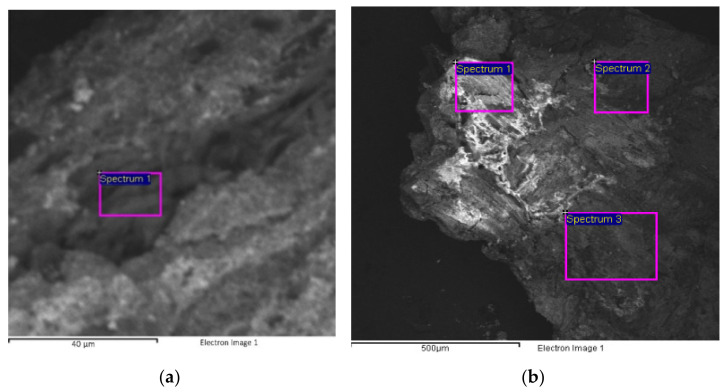
SEM images showing other details of sample 1. Image of the crystal-like dark contrasting area corresponding to the first paint layer (**a**) with the indication of the analyzed areas corresponding to the results in Table 3. Image of the paint layers (**b**) with the indication of the analyzed areas corresponding to the results in Table 4.

The morphological heterogeneity of the varnish is due to the texture of the paint layers: the more protruding strokes are not covered by the resin, which lies in the concave areas (Figure 45a,5b). In addition, the varnish is missing in the restored areas, where the yellow-green fluorescence is interrupted (Figure 46a,6b).

**Figure 7:**
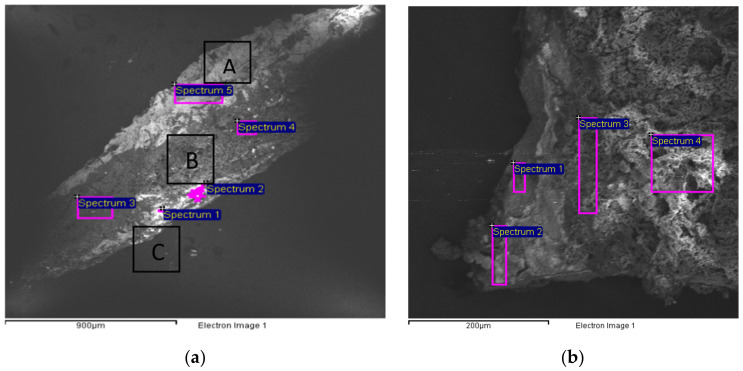
SEM images of samples 2 and 3. Image of sample 2 (**a**) with the indication of the different sections (A corresponds to the primer layer, B to the canvas, and C to the paint layer) and the analyzed areas corresponding to the results in Table 5. Image of the layers from the preparation layer to the paint layer (**b**) with the indication of the analyzed areas corresponding to the results in Table 6.

Eventually, the images acquired on the edge of the painting unearthed almost the whole stratigraphy of the artwork, showing the texture of the canvas, a white ground layer, thin blue paint layers, and, as already mentioned, a non-homogeneously spread resinous material (Figure 47a,7b).

### 3.3. Raman Spectroscopy

Raman spectroscopy was performed on four points of the painting, selected based on their significance, i.e., an area of the original canvas where both the ground and the paint layer were missing, a blue retouched spot with yellowish stains, the neck of the main figure, and the blue paint layer of the sky (Figure 1A–D). The collected spectra show high fluorescence interfering with the Raman signal and attesting the presence of an organic substance on the surface that cannot be detected by Raman spectroscopy. The reason for that is the inherent weakness of the Raman signal which relies on the weak process of inelastic light scattering. Thus, Raman’s detection is more challenging when organic materials are analyzed and fluorescence emission interferes [9]. The canvas also shows a noisy spectrum due to fluorescence emission, even though both the ground and the paint layers are missing in this area.

### 3.4. SEM/EDS

The backside of sample 1 was analyzed using the back-scattered electron detector to exploit the atomic number contrast making chemical elements with a higher atomic number appear in light-gray contrast. The resulting image clearly shows the weaving of the canvas with a yarns ratio of 1:1, and particles consisting of heavy metals such as Zinc (Zn), Barium (Ba), and Lead (Pb) (spectrum 1, Figure 5a, Table 1). All these elements belong to white pigments used in both the paint and the primer layers of oil paintings, namely lead carbonate [(PbCO3)2·Pb(OH)2], barium sulfate (BaSO_4_), and either zinc oxide (ZnO) or lithopone (BaSO_4_ and ZnS). The high amount of carbon (C) has to be related to the canvas.

Another area of sample 1 was investigated, revealing almost the entire stratigraphy of the painting on different levels (Figure 5b, Table 2). The arrow in Figure 5b begins by the paint layer passing through the primer layer up to the preparation layer. On the first layers, EDS analysis highlights the presence of sulfur (S), which can be related to both calcium (Ca) and barium (Ba) (spectra 1–2, Figure 5b, Table 2). Barium is generally present in the paint layers as barium sulfate (BaSO_4_), namely a white extender commonly used for pigments, or as lithopone (BaSO_4_ and ZnS). Indeed, synthetic barium sulfate was developed in the early 19th century. Its transparency made it a good extender for oil colors and as inert substrates for lake pigments, while lithopone is a white pigment produced since 1874 and widely used in ground layers or as a filler [8,15]. Calcium could be linked to the presence of calcium sulfate (gypsum, CaSO_4_), which has been commonly used as a preparation layer from early medieval times until the modern era [8,16]. The analysis also highlighted the presence of zinc and lead in the primer layer (spectra 1–3, Figure 5b, Table 2), confirming the use of lead carbonate (Lead White), and either zinc oxide (Zinc White) or lithopone. The counts of oxygen and carbon were removed from Table 2, Table 3, and Table 4, because they were detected in all the acquired spectra, even though in variable quantities. Therefore, they cannot be considered characteristic of any chemical species. C and O counts were maintained in Table 1 and Table 5 because the high amount of C is linked to the cellulose of the canvas. Spectra 1, 3, and 4 detected the presence of silica (Si), aluminum (Al), magnesium (Mg), and iron (Fe), suggesting the use of Green Earth in the first paint layers [17]. This could be partially responsible for the bluish-green color of the paint layer, together with the presence of chromium (Cr), which could be attributed either to Chromium Oxide Green, Viridian (hydrated chromium (III) oxide), or a mixture of Chrome Yellow and Prussian Blue, due to the presence of Fe [18]. Lead White is detected in higher amounts here, probably indicating that it was used mixed with the other pigments to obtain a lighter tone.

Sample 1 is characterized by a crystal-like dark area corresponding to the first paint layer (Figure 6a, Table 3). This was further investigated, revealing the presence of magnesium (Mg), silica (Si), and calcium (Ca), possibly identifying calcium and magnesium silicates. However, further investigation is needed to better understand the composition of this area.

The surface layer of sample 1 was also further examined to better characterize the bluish-green paint layer on the top (Figure 6b, Table 4). The presence of an organic substance emerged from the observation of the resulting image, showing a dark-grey contrast. Once again, EDS analyses detected the presence of magnesium (Mg), aluminum (Al), iron (Fe), and silica (Si), once again suggesting the use of Green Earth. The identification of chromium (Cr) can be related either to Chromium Oxide Green, Viridian, or Chrome Yellow mixed with Prussian Blue, due to the detection of Fe. They were presumably used in combination with Lead White, barium sulfate, and either Zinc White or lithopone.

The SEM image of sample 2 was divided into three sections to better understand the composition of each layer (Figure 7a, Table 5). EDS analysis confirmed that the white primer layer is composed of Lead White, identified by the presence of lead, and Zinc White, related to the detection of zinc. Indeed, if compared to sample 1, barium is missing, suggesting the presence of Zinc White instead of lithopone, while the simultaneous presence of sodium (Na), aluminum (Al), silicon (Si), sulfur (S), and oxygen (O) enabled the characterization of ultramarine blue as the inorganic pigment used for the blue background, as suggested also by the FT-IR ATR spectroscopy (Figure 7a, Table 5) [19,20,21]. Once again, the saturation of the blue layer was probably attenuated using Lead White and Zinc White (spectra 1–2, Figure 7a, Table 5). Eventually, the high amount of C in spectra 3 and 4 has to be related to the canvas. The same spectra also suggest the presence of a gypsum-based preparation (spectra 3–4, Figure 7a, Table 5).

SEM/EDS analysis of sample 3 shows the whole original stratigraphy, thus leaving out the varnish layer (Figure 7b, Table 6). Once again, the counts of oxygen and carbon were removed from Table 6 because they cannot be considered characteristic of any chemical species. It is possible to distinguish the canvas with a preparation layer probably consisting of gypsum mixed with zinc-based fillers (spectra 3–4, Figure 7b, Table 6). In addition, it is possible to hypothesize that the primer layer is composed of Lead White and Zinc White, due to the absence of Ba (spectrum 2, Figure 7b, Table 6). The paint layer of sample 3 shows high copper (Cu) content indicating the use of copper-based pigments that are also responsible for the blue color (spectrum 1, Figure 7b, Table 6).

### 3.5. FT-IR ATR Spectroscopy

**Figure 8:**
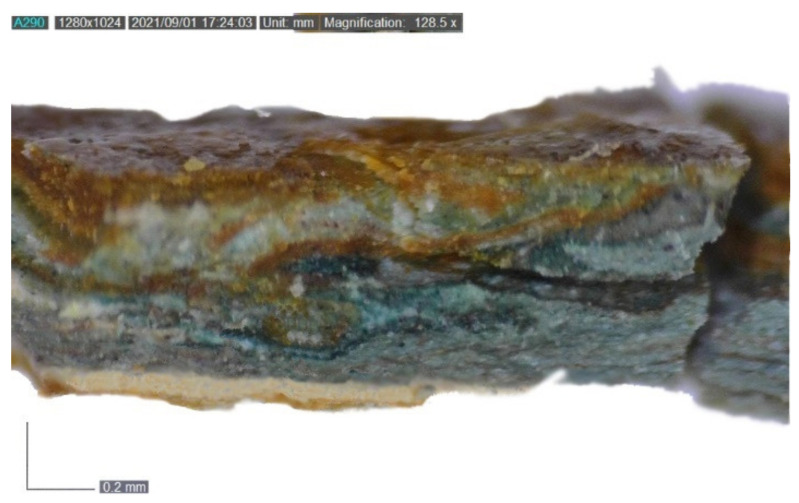
DinoLite image (128.5×) of a section of sample 1 before FT-IR ATR analysis. From the bottom to the top: canvas (partially visible), ground and primer layers, bluish-green paint layers, and varnish.

Before performing FT-IR ATR analyses, the samples were observed under the DinoLite portable digital microscope in both VIS and UV light at different magnifications (Figure 8). FT-IR ATR spectroscopy was carried out to obtain broader understanding of the materials constituting the stratigraphy. Samples were analyzed without pretreatment due to their tiny dimension.

**Figure 9:**
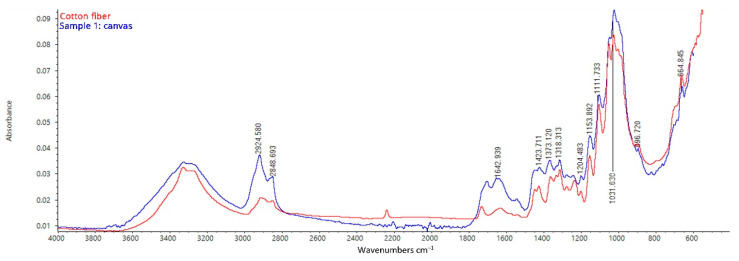
FT-IR ATR spectrum acquired on the backside of sample 1, consisting of the original canvas, the preparation layer, the primer, the paint layers, and the varnish: analysis of the backside of the canvas.

Spectra collected from the backside of the canvas (samples 1, 2, and 3) appear to be characterized by the typical cellulose bands of cotton or linen fibers, namely those at 3450–3320, 2925–2848, 1642, 1373, 1050, 1007, 897 cm^−1^ (Figure 9) [22]. In particular, the band near 897 cm^−1^ corresponds to the CH deformation of β-glycosidic linkages, which is related to the abundance of amorphous cellulose [23]. In addition, the peaks at 3450–3320 cm^−1^ (–O–H stretching), 2925 cm^−1^(–C–H stretching), 1642 cm^−1^ (–O–H bond vibrations including intermolecular hydrogen bonds between polysaccharide chains), and 1050 cm^−1^ (–C–O–C pyranose ring skeletal vibration) are characteristic of cellulose [24,25]. Even though cellulose fibers such as linen and cotton show similar FT-IR spectra, similarities with cotton fibers can be noticed in the acquired spectrum [26]. The hypothesis is supported by the spread production of cotton canvases in Italy at the beginning of the 20th century [27].

**Figure 10:**
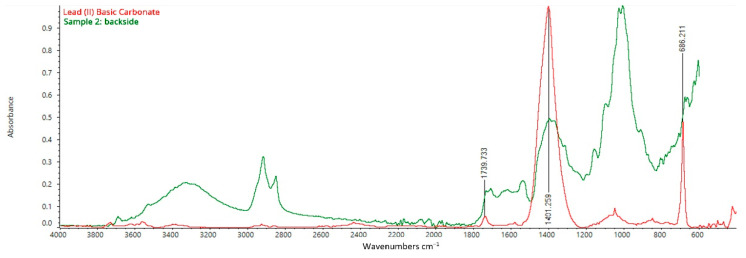
FT-IR ATR spectrum acquired on the backside of sample 2, consisting of the original canvas, the preparation layer, the primer, and the paint layers: analysis of the backside of the canvas.

In addition, an in-depth analysis of the spectrum collected from the backside of the canvas of sample n. 2 once again highlighted the presence of characteristic peaks which can be related to cellulose, but also to Lead (II) Basic Carbonate [(PbCO_3_)_2_·Pb(OH)_2_] (Figure 10) [28]. Bands at 1401 and 686 cm^−1^ identify C=O vibrations in the carbonate ion CO_3_^2−^, which can be attributed to the use of Lead (II) Basic Carbonate. In addition, the IR peaks at 1740 and 677 cm^−1^ are also typical of this compound. The presence of the so-called Lead White matches the dating of *I Tesori del Mare* because it has been used in almost every painting in Western art throughout the centuries until the first quarter of the 20th century [8]. Then, Zinc oxide (ZnO) gradually became to replace Lead White during the second quarter of the XIX century. Afterward, commercially produced Titanium White (TiO_2_) had begun to replace the toxic lead white pigment by the first quarter of the 20th century [8]. Eventually, the high peak at 1006 cm^−1^ identifies the presence of silica-based minerals such as kaolinite or ultramarine blue, while the presence of gypsum on the canvas detected by SEM/EDS analysis could not be confirmed by FT-IR ATR due to the thinness of the layer (Figure 10) [29,30,31].

**Figure 11:**
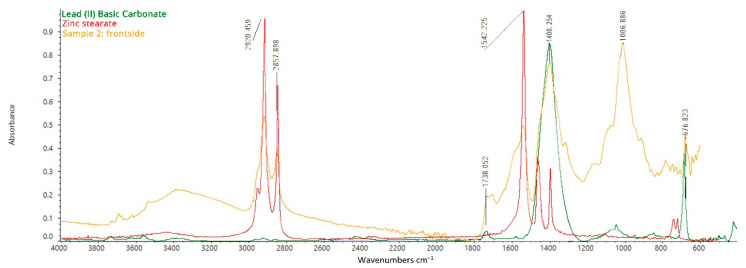
FT-IR ATR spectrum acquired on the front side of sample 2, consisting of the original canvas, the preparation layer, the primer, and the paint layers: analysis of the paint layer on the top.

Once again, spectra collected by placing both the preparation layer and the top layer of samples n. 1 and 2 on the ATR diamond show bands attributable to Lead (II) Basic Carbonate (Lead White) and metal soaps constituting the paint layer (Figure 11) [32,33,34,35]. The peak at 1542 cm^−1^ is related to the asymmetrical stretching vibrations of COO^−^ [36]. It can be attributed to the presence of metal soaps in the layer, which could either have been intentionally used as additives to modify the physical properties of the painting materials or be the result of chemical reactions between metal ions constituting the pigments and free fatty acids from lipidic binders (in this case, oil) [37,38,39,40]. In addition, the characteristic absorption band at 3565–3530 cm^−1^ is attributed to the vibrational motions of –O–H confirming the detection of both lead carbonate and linseed oil. The bands at 2960, 2855, and 1739 cm^−1^ are assigned to the vibrational asymmetrical stretching of –C–H_2_, vibrational symmetrical stretching of –C–H_2_, and –C=O of the triglyceride cross-linked network characteristic of siccative oils.

The peak at 1378 cm^−1^ corresponds to the wagging vibration of –C–H_2_, that at 1100 cm^−1^ to the asymmetrical stretching of –O–CH_2_–C, and that at 723 cm^−1^ to the rocking of –(C–H_2_)_n_– and the wagging of (C–H)=C–H, all confirming the presence of a siccative oil. The spectrum acquired on sample 2, canvas side, once again shows the high peak at 1006 cm^−1^, evidently related to silica-based pigments such as kaoline or ultramarine blue.

**Figure 12:**
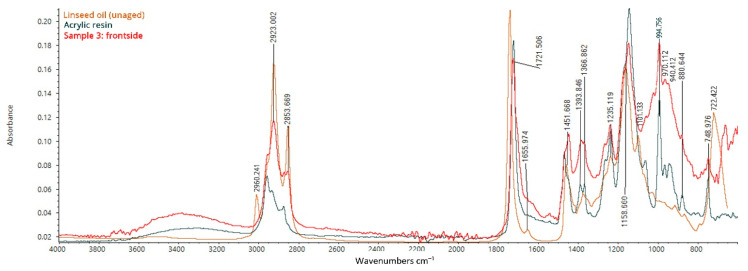
FT-IR ATR spectrum acquired on the front side of sample 3, consisting of the original canvas, the preparation layer, the primer, the paint layers, and the varnish: analysis of the frontside (paint layers and varnish).

The spectrum collected on the front of sample 3 was compared with unaged linseed oil and an acrylic resin used as a finishing and retouching varnish (Figure 12). The spectrum shows the characteristic bands of both aged linseed oil and acrylic resins (Figure 12).

The peaks at 2931 and 2853 cm^−1^ (–C–H stretching) are related to the presence of aged linseed oil, which differs from the raw type for the missing peak at 2960 (–C–H stretching), 1101 (–C–O stretching), and 722 cm^−1^ (–C–H bending), and for the appearance of the peaks at 1394 (–C–H bending) and 1367 cm^−1^ (–O–H bending) [35]. Peaks that are common to either aged linseed oil or acrylic resin are those at 1721, 1451, 1394, 1367, 1235, 1159 cm^−1^, while the bands at 1270, 994, 970, 940, and 880 cm^−1^ seem to be related to the acrylic resin alone [41,42]. Eventually, the analysis further confirms both the use of linseed oil as a binder and the presence of a non-original layer presumably applied during past restoration treatments.

Attempts were made to gain further information about the intermediate layer by placing the three samples sideways on the diamond of the ATR. The spectra collected showed similar peaks to the already analyzed paint layers.

## 4. Discussion

The implemented multi-analytical approach enabled the acquisition of relevant data regarding the constituting materials and the causes of degradation of *I Tesori del Mare* (1901) by Plinio Nomellini. At the time of the analyses, the oil painting on canvas showed consistent liftings and alteration of the varnish layer, requiring in-depth investigation to guide the conservation treatment. The joint use of both non-invasive and micro-invasive diagnostic techniques provided significant information while preserving the artwork’s integrity. Multispectral imaging allowed for the acquisition of the first data regarding the fluorescence of the varnish and the paint layer on the top, also unearthing the structure of the figuration. DinoLite digital microscopy enabled identification of the restored areas and better understanding of the varnish distribution, which appeared to be unevenly spread on the entire surface of the painting. In addition, it allowed for a more detailed observation of the color gaps, providing first details of the painting’s stratigraphy. Results obtained using Raman spectroscopy were not able to provide relevant data due to the high fluorescence which interfered with the Raman signal, attesting the presence of an organic substance.

FT-IR ATR and SEM/EDS analyses provided more detailed information on the pigments and the causes of the paint layer’s liftings. Zinc appears to be present in the preparation layer (in the form of zinc oxide or lithopone)—probably mixed with gypsum—the primer and the paint layers (probably in the form of zinc oxide). In addition, FT-IR ATR spectra detected peaks related to metal soaps in the hole stratigraphy besides the canvas. They could either have been intentionally added to modify the mechanical properties of the paint or be the consequence of the reaction between the pigments’ metal ions and the binder [40]. Zinc soaps are known to be more likely associated with degradation phenomena than lead soaps, affecting paint layers’ delamination and flaking [43,44,45,46]. Thus, it is reasonable to assume that the cyclic and persistent occurrence of liftings and gaps of the paint layer, which required frequent fixing intervention during the years, are caused by of the presence of zinc soaps in all the stratigraphy.

Lead (II) Basic Carbonate (Lead White) was found to be one of the major components of both the primer and the paint layers, respectively, being used as a pigment and whitening agent, like Zinc White. In addition, elements that could be related to pigments such as Green Earth, Chromium Oxide Green, Viridian, Chrome Yellow mixed with Prussian Blue, Ultramarine Blue, but also copper-based and silica-based pigments, were detected in the blueish paint layer, being responsible for the blue-green hue. The exact identification of pigments could have been sustained by micro-Raman and FT-IR analyses performed on the samples embedded in resin. However, in this circumstance only three tiny samples were taken to preserve the artwork integrity, thus hindering the possibility of resin-embedding and opening up different hypotheses on the pigments’ characterization.

Eventually, FT-IR ATR spectroscopy highlighted the possible use of a cotton canvas and the presence of linseed oil as the binder for the pigments, which shows an advanced oxidative state. In addition, it enabled the identification of an acrylic resin commonly used as retouching or finishing varnish applied on the paint layers. It appears to be unevenly spread on the paint layer due to both the presence of brushstrokes in relief and the frequent restoration treatments that the painting underwent during the years.

## 5. Conclusions

Images acquired using both multispectral imaging and portable digital microscopy outlined the remarkable complexity of the artwork, which is composed of multiple superimposed layers. The combined use of non-invasive and micro-invasive analyses clarified the artistic technique and the nature of part of the pigments used by the artist, which appear to comply with the dating of the artwork. In the analyzed areas, the painting appears to be composed of the following layers:

- Canvas fabric, presumably made of cotton fibers.
- Preparation layer, presumably consisting of calcium sulfate (gypsum) mixed with Zinc White particles or lithopone.
- Primer layer made with linseed oil (currently, in an advanced oxidized state), Lead White and probably Zinc White with traces of lithopone deriving from the underlying layer.
- Blueish paint layer, consisting of linseed oil (currently, in an advanced oxidized state), Zinc White, Lead White, and pigments responsible for the blue-green hue (presumably, Green Earth, either Chromium Oxide Green, Viridian, or Chrome Yellow, mixed with Prussian Blue, Ultramarine Blue, copper-based pigments, and silica-based pigments).
- Varnish layer, made of an acrylic resin commonly used as a retouching and finishing varnish.

Eventually, the analyses allowed for deep understanding of the causes of deterioration. Since its creation, the painting *I Tesori del Mare* has cyclically showed color gaps and flaking paint, constantly requiring fixing of the paint layer. The joint use of the implemented analytical techniques enabled the identification of Zinc and linseed oil as some of the major components of the stratigraphy. Zinc-based pigments could have readily reacted with fatty acids present in oils to form degradation products such as zinc stearate and zinc palmitate, resulting in severe delamination and brittleness of the paint layers. However, metal soaps could have also been intentionally added to the paints during the execution stage to modify the physical properties of the paint layers. Either way, the degradation phenomena are rooted in chemical processes inherent to the primer and the paint layers, resulting in overall embrittlement and instability of the painting.

## Figures and Tables

**Figure 4:**
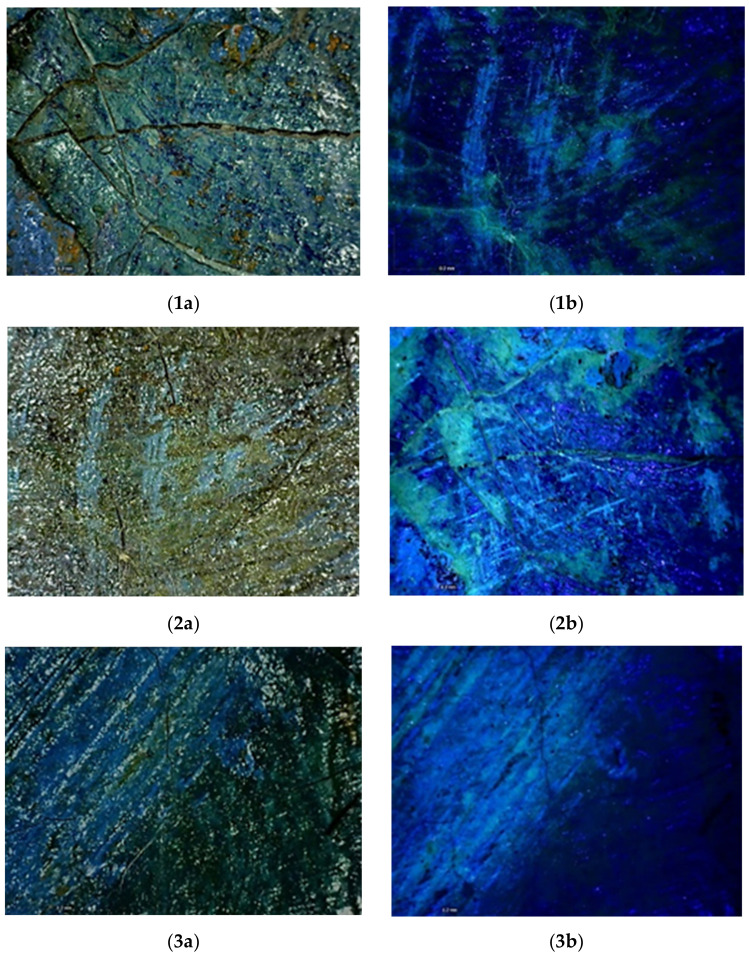

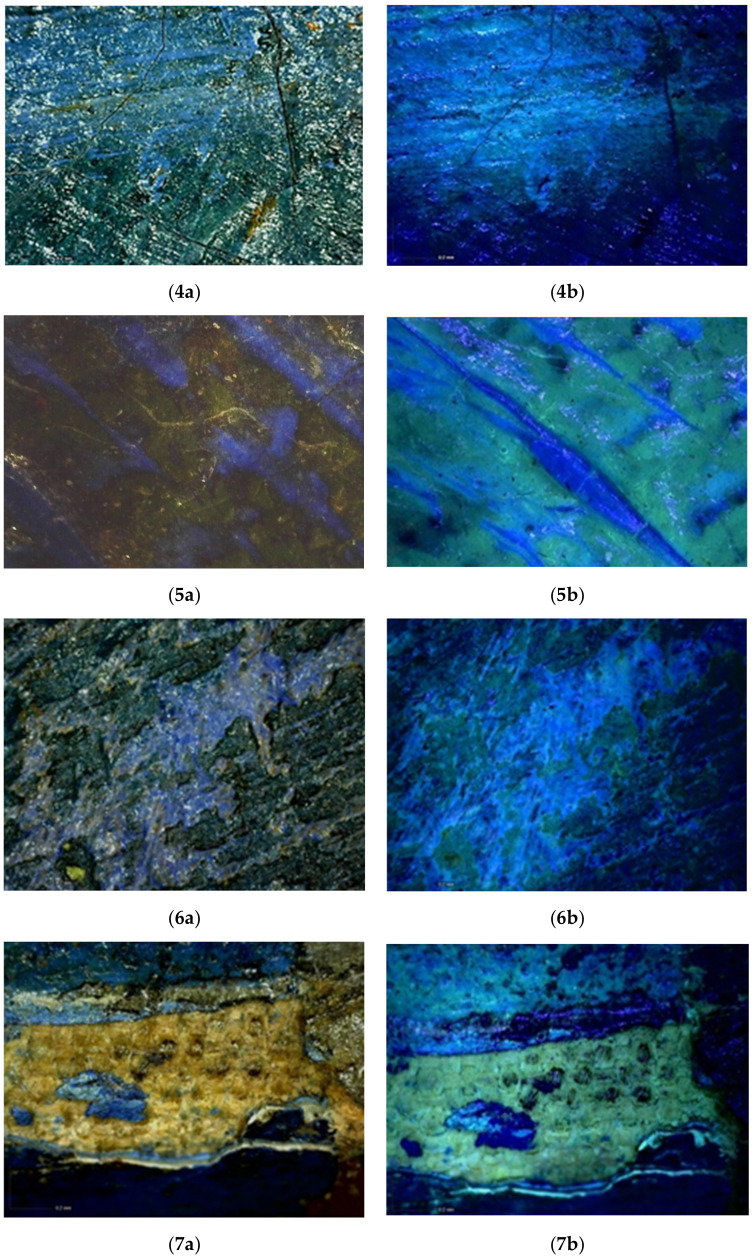
DinoLite images (50× enlargements) with numbers in the caption corresponding to the map in Figure 1. Details of the lifted areas in VIS (**1a**) and UV light (**1b**). Details of the varnish and the paint layers in VIS (**2a**–**5a**) and UV light (**2b**–**5b**). Details of the restored areas (chromatic reintegration) in VIS (**6a**) and UV light (**6b**). Details of the color gap, the canvas, and part of the overlying layers in VIS (**7a**) and UV light (**7b**).

**Table 1:** Elements detected by EDS analysis of the areas shown in Figure 5a. Results are reported in weight percentage (w%).

Spectrum	C	O	Na	Si	S	Ca	Fe	Zn	Ba	Pb
Spectrum 1	27.8	26.2		2.0	3.6		10.0	13.0	11.4	6.0
Spectrum 2	45.0	35.3		2.3						17.4
Spectrum 3	55.0	45.0								
Spectrum 4	53.6	46.0				0.4				
Spectrum 5	48.4	50.4	0.5	0.2	0.3	0.2				

**Table 2:** Elements detected through EDS analysis of the areas shown in Figure 5b. Results are reported in weight percentage (w%) removing the counts of oxygen and carbon.

Spectrum	Mg	Al	Si	S	K	Ca	Cr	Fe	Zn	Ba	Pb
Spectrum 1	8.2	7.8	17.8	5.6	1.4	3.2	2.8	8.0	12.0	9.2	24.0
Spectrum 2		6.8	15.3	8.0		2.4	1.4	19.3	7.8	19.0	20.0
Spectrum 3	14.4	6.0	21.6			1.6	0.9	4.0	11.0	3.0	37.5
Spectrum 4	4.9	7.8	12.3			1.3	2.6	2.6	14		54.5
Spectrum 5		9.8	12.3			1.6	0.2	2.2	2.8		71.3

**Table 3:** Elements detected through EDS analysis of the areas shown in Figure 6a. Results are reported in weight percentage (w%) removing the counts of oxygen and carbon.

Spectrum	Mg	Al	Si	Ca	Fe	Zn	As
Spectrum 1	11.2	0.8	12.4	4.1	0.4	0.6	0.3

**Table 4:** Elements detected through EDS analysis of the areas shown in Figure 6b. Results are reported in weight percentage (w%) removing the counts of oxygen and carbon.

Spectrum	Mg	Al	Si	S	Ca	Cr	Fe	Zn	Br	Ba	Pb
Spectrum 1		8.6	15.7	19.6	5.8		19.0	7.7		23.6	0.00
Spectrum 2	5.2		11.2	8.2	3.0		21.6	17.2	trac	19.4	14.2
Spectrum 3	8.6	8.7	19.6	11.2	4.7	2.6	13.3	22.0		9.3	0.00

**Table 5:** Elements detected through EDS analysis of the areas shown in Figure 7a. Results are reported in weight percentage (w%).

Spectrum	C	O	Na	Al	Si	S	Ca	Fe	Zn	Pb
Spectrum 1		54.0	10.5	7.6	8.0		0.8		2.4	16.7
Spectrum 2	58.0	28.7		0.8	1.2		0.7	0.7	2.6	7.3
Spectrum 3	41.4	48.3			0.2	5.2	4.9			
Spectrum 4	55.3	37.2			0.9	2.0	3.0	1.7		
Spectrum 5		44.4		2.4	1.7		1.4			50.0

**Table 6:** Elements detected through EDS analysis of the areas shown in Figure 7b. Results are reported in weight percentage (w%) removing the counts of oxygen and carbon.

Spectrum	Al	Si	S	Cl	K	Ca	Ti	Fe	Cu	Zn	As	Pb
Spectrum 1	7.8	14.8	2.8	1.0	3.2	1.2	1.4	4.6	14.6	44.8	3.8	
Spectrum 2	4.7	11.2			1.6	5.0		4.4	2.8	21.0		49.3
Spectrum 3		1.7	38.7			43.4		4.0		7.0		5.2
Spectrum 4		3.3	41.3			52.2				3.2		

## Data Availability

Publicly available datasets were analyzed in this study to compare the acquired FT-IR spectra with already available spectra of standard materials. Data can be found here: https://spectra.chem.ut.ee/ (accessed on 9 April 2022).
